# Experimental evaluation of the effect of openings on the structural performance of diagonally reinforced coupling beams

**DOI:** 10.1038/s41598-025-25787-7

**Published:** 2025-12-15

**Authors:** Ahmed Elghool, Nasser Elshafey, Osman M. O. Ramadan, Mohamed S. Elbayomy

**Affiliations:** 1 Department of Structural Engineering, Cairo University, Cairo, Egypt; 2 Department of Structural Engineering, The Higher Institute of Engineering and Technology Fifth Settlement, Cairo, Egypt

**Keywords:** Civil engineering, Engineering

## Abstract

In some cases, it is necessary to create openings in coupling beams to accommodate utility pipes and ducts. The presence of these openings can alter the behavior of coupling beams, making their performance differ from that of solid coupling beams (without openings). However, research on the structural performance of coupling beams with openings is limited. To address this gap, three diagonally-reinforced half-scale specimens were investigated in this study, two with openings near the span end of coupling beams and one without (solid) which served as the reference. For one sample, the use of additional diagonal bars at opening corners together with short stirrups above and below the openings were investigated as an attempt to mitigate the effect of opening. The results should help design engineers to understand the behavior of coupling beams containing openings and give solution to mitigates the negative effect of openings when necessary. Based on the conducted experiments, it is shown that the presence of openings has a pronounced effect on the overall behavior of coupling beams. Adding an opening with relative dimensions of *(l*_*o*_*/l* = 0.14 and *h*_*o*_*/t* = 0.24) near the end span of the beam increased the number and width of cracks, and reduced both the beam strength and ductility by 16.25% and 20.30%, respectively, compared to the corresponding solid coupling beam. Nevertheless, reinforcing the opening with diagonal bars as well as using short stirrups in beam parts above and below the opening mitigated the negative effects of the opening presence. Specifically, this added reinforcement controlled cracking, and increased beam strength and ductility by 10.46% and 23.60%, respectively. This brought the beam behavior closer to that of a solid one (without openings). It is also found that the ACI-318’s shear capacity equation for diagonally reinforced coupling beams is conservative and needs to be modified. The later findings agree with a number of previous studies.

**Supplementary Information:**

The online version contains supplementary material available at 10.1038/s41598-025-25787-7.

## Introduction

**Fig. 1:**
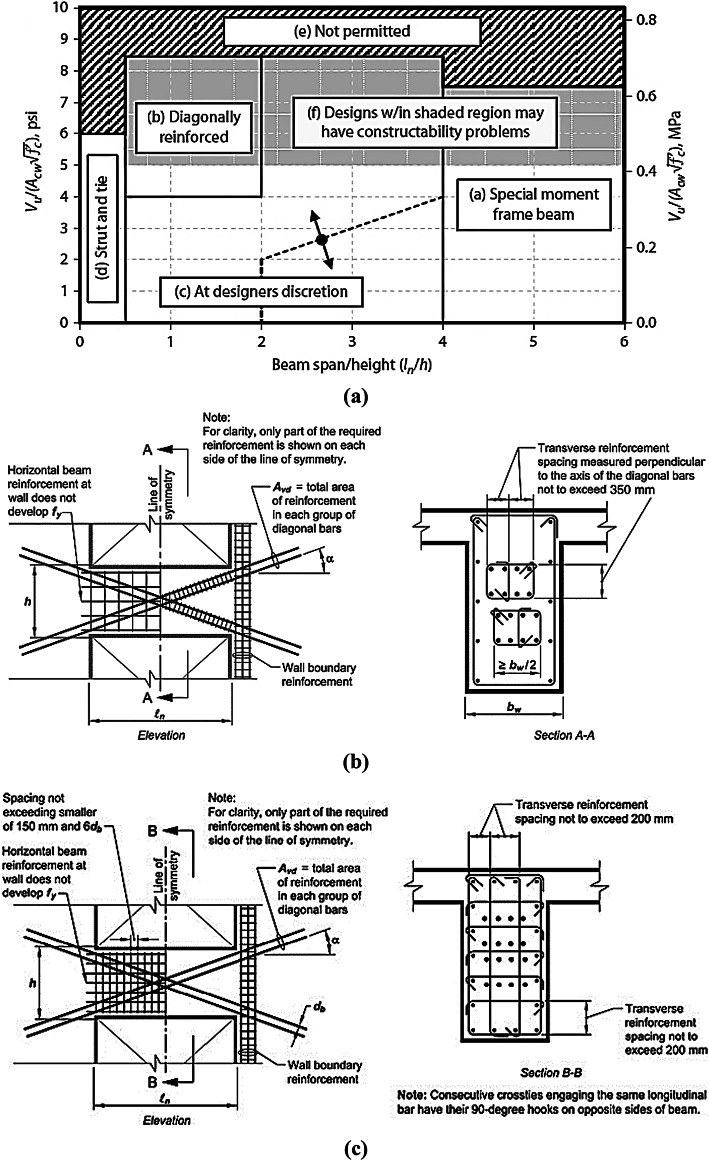
Coupling beam design guidelines as per ACI code. (a) Guidelines for chosen RFT details of coupling beams; (b) confinement of individual diagonals; and (c) full confinement of beam section.

Coupling beams (CBs) are important structural members that act as fuses in coupled shear-wall seismic load-resisting systems. As such, precautions for their design and detailing have been recommended by various codes and research studies. Two types of CBs are recommended in^1^, based on the relationship between the beam span and its depth (*l*_*n*_*/h*) and the actual shear stress, as illustrated in Fig. 1a. For coupling beams with (*l*_*n*_*/h* > 4), using reinforcement (RFT) detailing with top and bottom bars (conventional detail) is acceptable. However, for coupling beams with (*l*_*n*_*/h <* 2), it is recommended to use two intersecting cages of symmetrical diagonal reinforcement (diagonal coupling beams) to prevent shear sliding failure. For coupling beams where (2 *< l*_*n*_*/h <* 4), the designer has the option to choose between conventional or diagonal CBs.

For diagonally reinforced coupling beams (DCBs), providing diagonal cages confined by ties, as shown in Fig. 1b, is recommended. However, this approach leads to construction difficulties, especially at the intersection location at mid-span. As a result, a new alternative option was proposed, which involves full confinement of the diagonally reinforced concrete beam section with a specified distance, as shown in Fig. 1c.

**Fig. 2:**
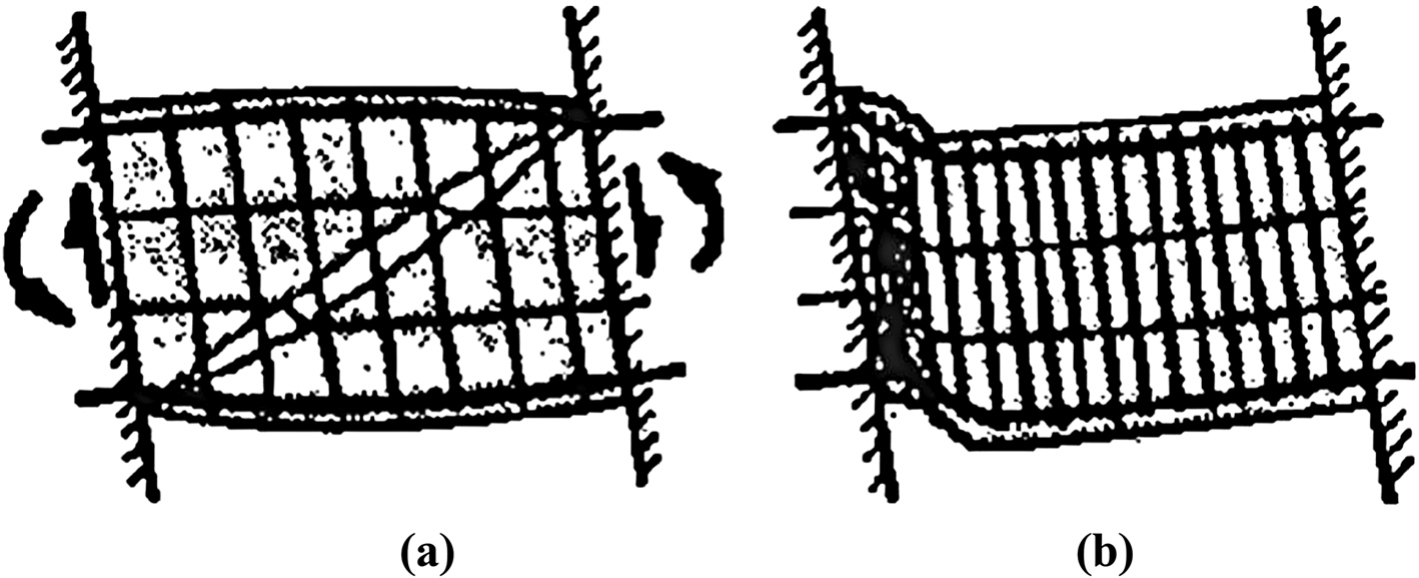
Failure modes of RCCBs (adopted by Paulay and Priestly, 1992): (a) diagonal compression failure; and (b) sliding failure.

Many practical and theoretical studies have been conducted to examine the behavior of coupling beams with traditional details (conventional and diagonal), including^2–14^. Based on the results of^2–7^, coupling beams with a diagonal scheme—whether confined around the diagonal or across the entire beam cross-section—exhibit more stable hysteretic performance, along with higher ductility, strength, and energy dissipation compared to corresponding solid coupling beams with conventional detailing, particularly for slender coupling beams. However, the diagonal detail with confinement stirrups can be difficult to construct due to reinforcement congestion. Paulay and Priestly^8^ identified two failure modes for CBs: the first is sliding shear failure, resulting from the interaction of shear and moment at the beam ends; the second is diagonal compression failure, caused by buckling of the diagonal bars and crushing of the concrete in the diagonal direction of the coupling beam, as shown in Fig. 2.

According to the experimental results of^7,12^, and^13^, the effect of confinement stirrups must be considered during the design process, as they significantly affect the overall behavior of coupling beams, including shear capacity, maximum chord rotation, and energy dissipation. These properties decrease as the spacing of the ties around the entire coupling beam increases. Numerous experimental studies, such as those by^3,5–7,10^, and^14^, suggest that the shear capacity formula for diagonally reinforced coupling beams recommended by the ACI 318^1^, which relies only on the diagonal bars for resisting shear, is too conservative and should be modified to include the contributions of concrete, stirrups, and longitudinal bars. Based on the results of^5,6,9,10^, and^11^, coupling beams tested with significantly higher strength values than the upper shear capacity limit recommended by the ACI 318^1^ (0.83$\sqrt {{f_c}^{\prime }}$) showed stable hysteretic performance and maximum chord rotations exceeding 5%, which is superior to the limiting value of *θ*_*u*_ specified in ASCE 41 − 17^15^ (*θ*_*ASCE*_ = 5%). Thus, the upper limit of strength is more conservative and should be modified.

1 $${V_{ACI}}=2{A_{vd}}{f_{yd}}\sin \alpha \leqslant 0.83\sqrt {{f_c}^{\prime }} {A_{cw}}$$

Many researchers, including^6,13,16,17^, have proposed new formulas to more accurately predict the shear capacity of diagonally reinforced coupling beams, addressing the shortcomings of the ACI-318’s formula. Other researchers have also proposed plastic hinge models for nonlinear analysis based on the shear mechanism of RC coupling beams^18–21^.

In addition, other researchers have investigated alternative reinforcement schemes to improve the seismic response and address the challenges faced by coupling beams with diagonal reinforcement. Studies have explored coupling beams with rhombic reinforcement schemes^3^, truss reinforcement bar details^22^, conventional details with intermediate horizontal bars and U-type reinforcement^23^, and double-beam coupling beams (DBCB) with and without keyways^24,25^. The results showed that all of these details provide better performance, ductile behavior, and reduced reinforcement congestion, except for the beam with a truss detail, which encountered some construction difficulties and failed to prevent sliding failure (brittle failure). This may have been due to the use of very narrow-width beams in the study.

Few studies, such as^26^, have investigated the behavior of coupling beams containing openings. This study included four short diagonally reinforced coupling beams (DCCBs) with a span-to-depth ratio (*l/h* = 1.29), three of which contained openings and one was used as a reference. The main parameters were the number and location of openings (top and bottom openings at mid-span or center openings at the two ends of the CBs). The openings were square-shaped (60 × 60 mm) with dimensions (*l*_*o*_*/l* = 0.13 and *h*_*o*_*/t* = 0.17). The results indicated that coupling beams with two openings at the mid-span, top and bottom, had a slight effect on the behavior of the beams compared to the solid ones. However, providing openings at the ends of the coupling beams, at the center height of the beam, decreased the stiffness and shear capacity by approximately 8.5% and 8.4%, respectively.

## Research significance

Although many researchers have investigated the behavior of coupling beams, few have examined those with openings. Additionally, there are no specific recommendations in codes regarding the behavior of coupling beams that contain openings. Therefore, this study aims to provide insights into the behavior of coupling beams with openings; and to identify the appropriate conditions for using openings in coupling beams. Thus, the study experimentally investigates the behavior of coupling beams with openings. It focuses on the effects of openings on important behavior parameters such as crack pattern and propagation, yield and ultimate strength, ductility, stiffness degradation, and energy dissipation. These parameters are compared to those of the corresponding solid beam. For one sample, intersecting diagonal bars around the opening and short stirrups above and below the opening, as recommended by Mansur (2006)^27^, were provided as an attempt to reduce stress concentration in the opening area and prevent premature cracking and failure.

### Experimental program

Three short half-scale coupling beams were prepared and tested in this study: two with openings (E-Op and E-Op-Ad) and one without (S), which served as the reference. The labels for the beams are as follows: " E-Op” refers to an opening near the span end and “Ad” refers to additional reinforcement. All specimens were designed to have a shear stress (q) equal to 0.48 $\sqrt {{{f^{\prime}}_c}}$ (MPa), where q is calculated as the shear strength of a coupling beam divided by its cross-sectional area (*b × h*). The shear strength (q) of the coupling beams was calculated according to Sect. 18.10.7.4 of ACI 318 − 19^1^.

**Fig. 3:**
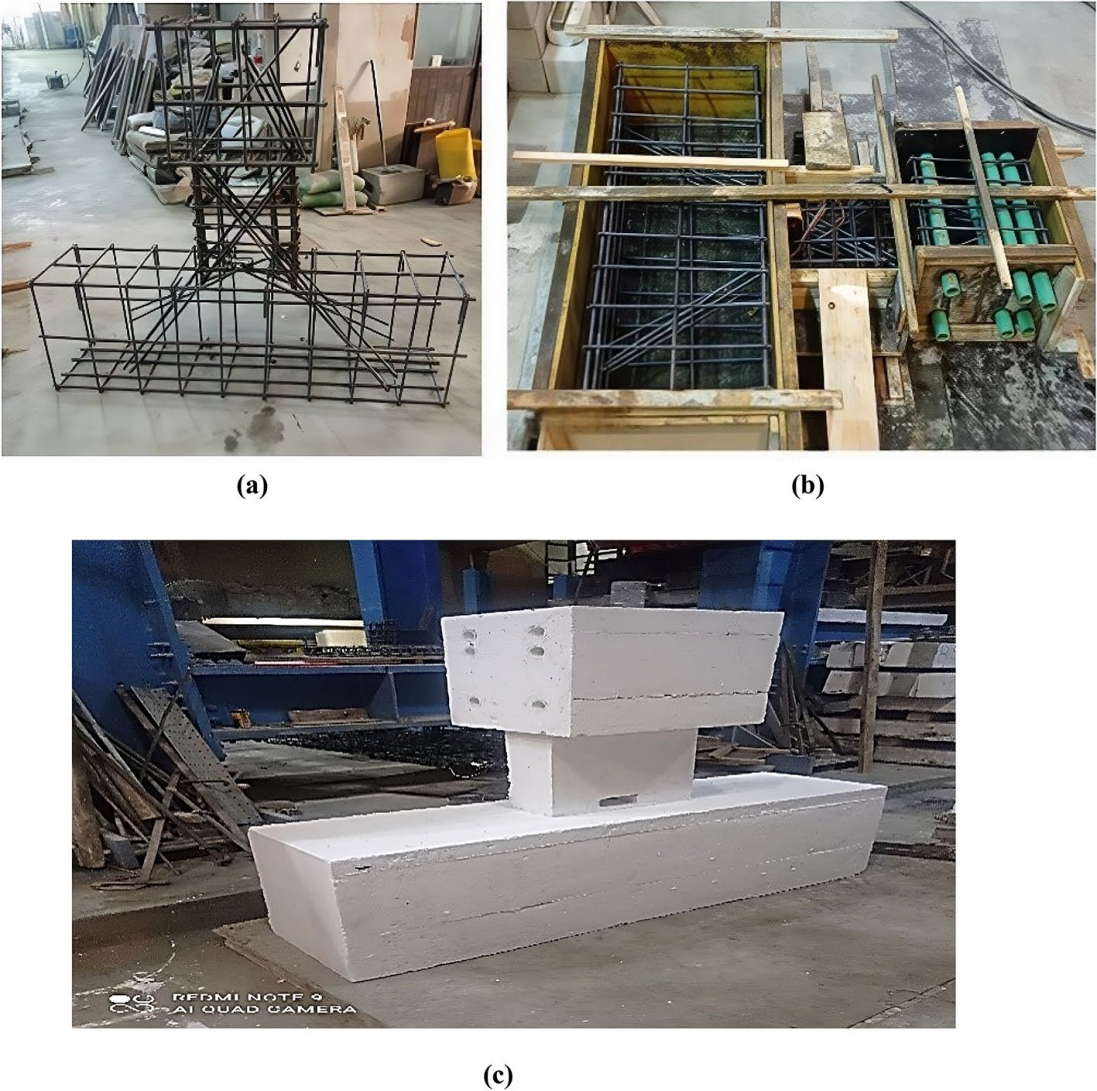
Preparing of specimens; (a) RFT cages; (b) ready for casting; and (c) after casting.

**Fig. 4:**
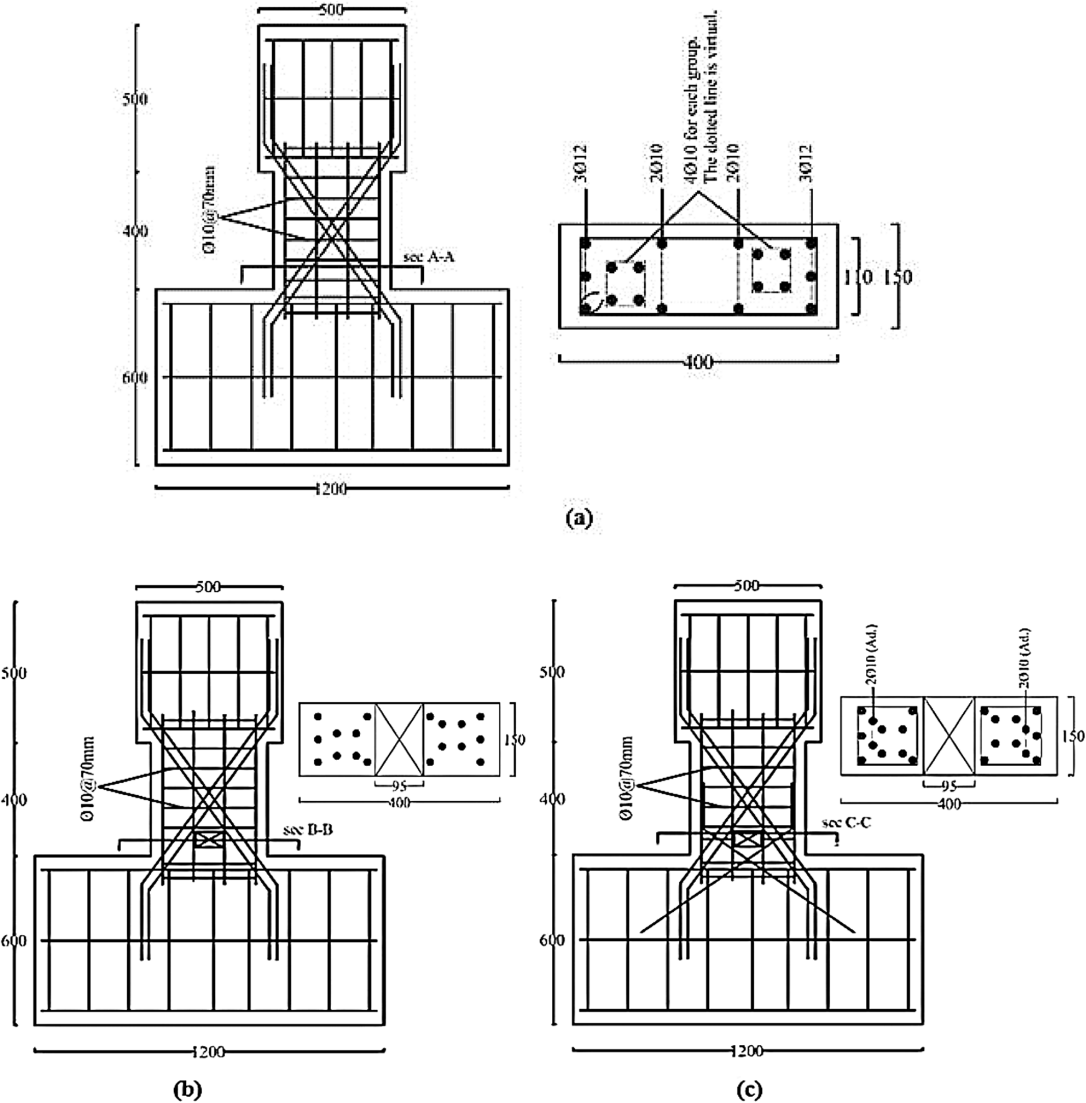
Geometry and details of RFT for specimens: (a) Specimen (S); (b) Specimen (E-Op); and (c) specimen (E-Op-Ad).

All specimens had the same dimensions: 400 mm for both depth and length (span-to-depth ratio, *l/h* = 1), and 150 mm for the width. The reinforcement details were constant for all specimens, featuring two diagonally reinforced cages with confinement stirrups around the interior beam cross-section, as permitted by the second option of the ACI 318^1^. The diagonal bars used were 4Ø10 for each group, with an inclination angle (*α* = 35°). Longitudinal bars were 3Ø12 at the top and bottom, with intermediate bars of 2Ø10 every 110 mm, and vertical stirrups of Ø8 every 70 mm, computed according to ACI recommendations^1^. The E-Op-Ad specimen differs by the addition of short stirrups and additional diagonal reinforcement above and below the opening. The opening located near the beam end (at distance of 30 mm from the fixed support). The opening dimensions were the same for the specimens with openings: 55 mm in width and 95 mm in height (*l*_*o*_*/l* = 0.14 and *h*_*o*_*/t* = 0.24). Specimens’ details, including opening locations, are summarized in Table 1. Besides, Figs. 3 and 4 show photos during specimen preparation with full beam layout and reinforcement details.

**Table 1:** Description of samples.

Beam name	Opening location in longitudinal direction of the beam (S)	Opening location in transverse direction of the beam (Z)	Description
S	―	―	Diagonally reinforced beam without diagonal confinement
E-Op	Spacing of 30 mm from the fixed support	At the mid height	
E-Op-Ad	Spacing of 30 mm from the fixed support	At the mid height	Diagonally reinforced beam without diagonal confinement
			With additional reinforcement around the opening

### Material properties

**Fig. 5:**
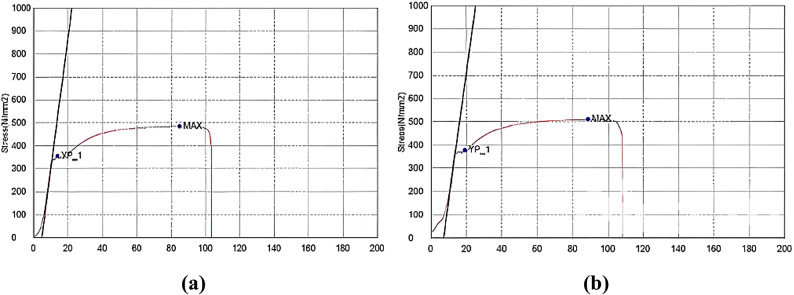
Stress-strain curve of steel reinforcing bars; (a) D10, and (b) D12.

A concrete mixture with a target compressive strength of 30 MPa was designed for coupling beams and blocks. Ordinary Portland cement Type 42.5 N was used, and the average water-cement ratio (*w/c*) was 0.50. Three cubes were prepared and tested at 28 days. The resulting cubic compressive strength ranged from 29 to 35 MPa, which was considered acceptable compared to the target design strength of 30 MPa. The ultimate compressive strengths for the specimens are listed in Table 2. Tensile tests were conducted on reinforcing (RFT) bars, and the resulting stress-strain curves are presented in Fig. 5. The bars have an elastic modulus of 200,000 MPa and a Poisson’s ratio of 0.3. The average yield strengths were 350 MPa (D10 bars) and 380 MPa (D12 bars), and the average ultimate strengths were 484 MPa (D10 bars) and 510 MPa (D12 bars).

**Table 2:** Ultimate cubic compressive strength of the tested specimens.

Specimen	S	E-Op	E-Op-Ad
*γ*_*c*_ (kN/m^3^)	26.97	26.8	27.85
*f*_*cu*_ (MPa)	31	29	35

### Test setup

**Fig. 6:**
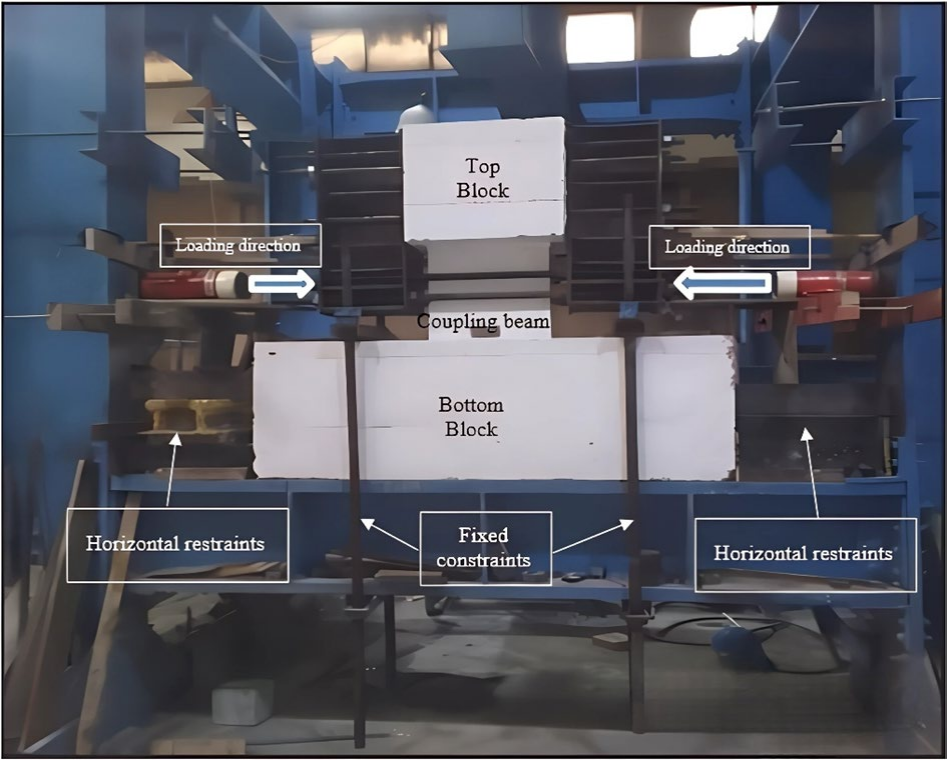
Test setup.

Two stiff blocks were used at the ends of coupling beams to simulate the surrounding walls; they also served in applying the load. The specimens were tested vertically; the lower block was restrained from movement using high-grade threaded rods and side constraints. The upper block was connected to a rigid steel system consisting of two I-beams (HEA300) with added web stiffeners and two 15 mm thick steel plates (one on each side of the upper block). The entire system (coupling beam, steel plates, and I-beams) was interconnected by high-grade threaded rods passing through pipes embedded in the upper block’s cross-section. The rigid steel system was designed to transfer the load along a line of action passing through the mid-span of the coupling beam, ensuring zero moment. The number and diameter of the high-grade threaded rods were designed to prevent buckling failure and ensure that all parts acted as a single rigid system. The rigid steel system was designed to transfer the entire load to the coupled wall system without any local deformation in the I-beams. The upper support acted as a free end condition without any restraints. Figure 6 illustrates the test setup and the position of each component.

### Methodology and instrumentation

**Fig. 7:**
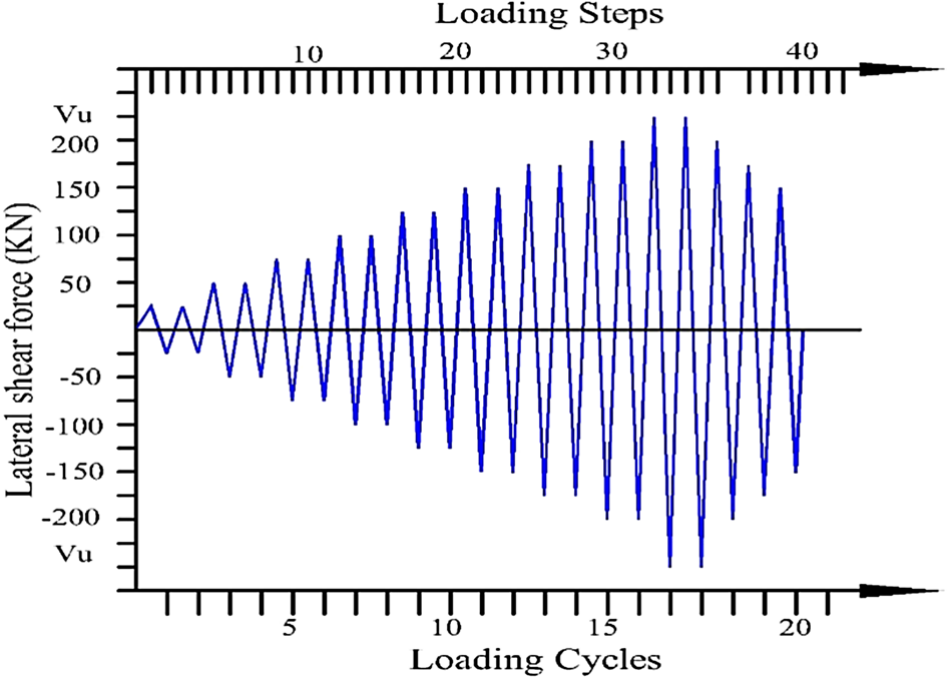
Historical cyclic load.

All specimens were subjected to the same quasi-static loading protocol to simulate the near-collapse mechanism of coupling beams in coupled shear wall systems subjected to earthquake forces. For each chord rotation amplitude, two consecutive loading cycles were applied to monitor the degradation in the coupling beam’s strength and stiffness. Force-controlled analysis was employed in this study despite the advantages of displacement-controlled loading for post-yield characterization, as the necessary equipment was unavailable. A constant loading rate of 15 kN/min was applied. To mitigate inertial effects, the load and deformation were closely monitored using strain gauges, displacement transducers, and load cells, enabling real-time adjustments to the loading rate to maintain quasi-static conditions. The load cycles increased gradually in 25 kN increments until significant crack widening indicated the onset of collapse and large displacements were recorded. The force was then gradually reduced to 0.80 of the ultimate force. The loading history is illustrated in Fig. 7. However, this type of loading differs from actual seismic response, which involves unsymmetrical loading and a fewer number of cycles.

**Fig. 8:**
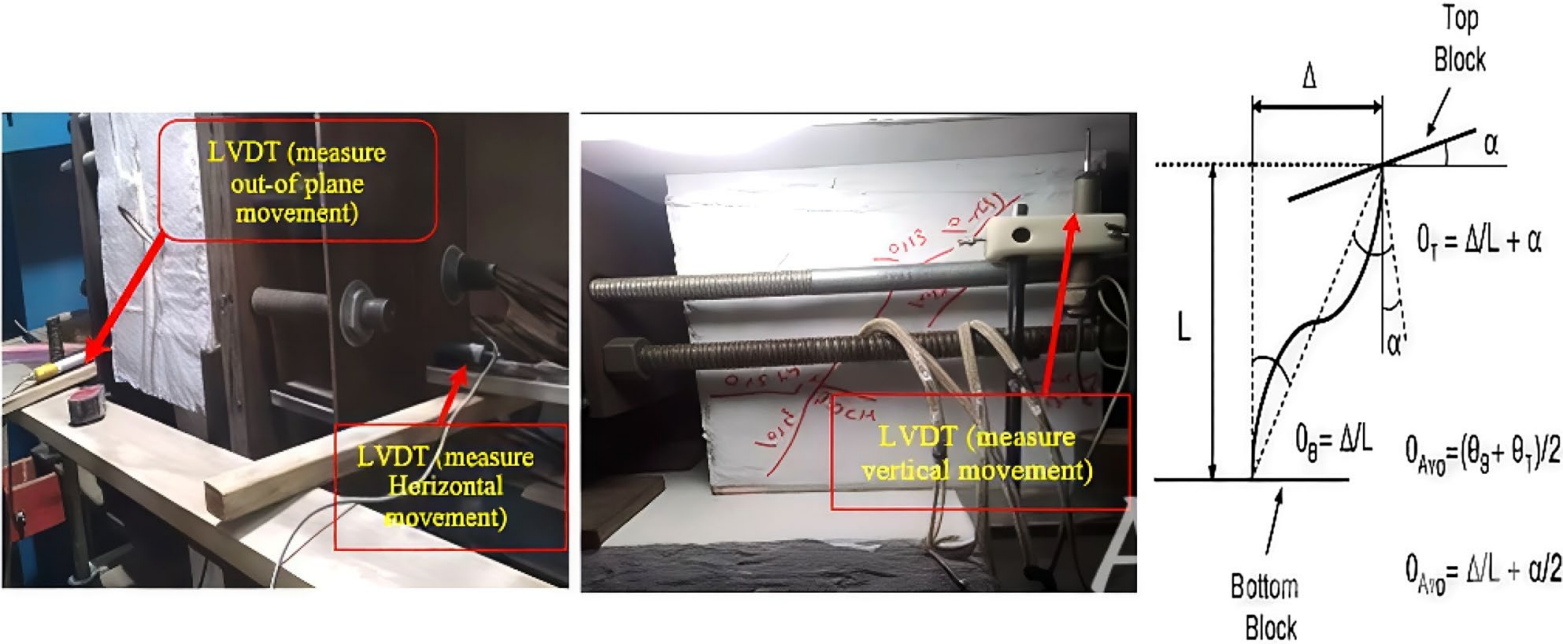
LVDT setup location and chord rotation measurement.

Based on the expected deformation of the coupling beams, see Fig. 8, two linear variable differential transducers (LVDTs) were installed at the right and left sides of the upper end of the beam to measure the positive and negative lateral displacements (*Δ*_*h*_). Since the upper block was unrestrained against rotation, one LVDT was used to calculate the vertical displacement (*Δ*_*v*_). Thus, the chord rotations at the bottom and top ends (*θ*_*b*_ and *θ*_*t*_) of the beam can be calculated as follows:

2 $${\theta _B}={\Delta _h}/{\text{ }}l$$

3 $${\theta _T}=\alpha \,+\,{\Delta _h}/{\text{ }}l$$

where:

4 $$\alpha ={\Delta _v}/{\text{ }}(h/2)$$

where; *l* and *h* are the beam length and height, respectively. Thus; the average chord rotation (*θ*_*Avg*_*)* can be computed as:

5 $${\theta _{Avg}}=({\theta _B}\,+\,{\theta _T})/2$$

6 $${\theta _{Avg}}={\text{ }}({\Delta _h}/{\text{ }}l\,+\,\alpha \,+\,{\Delta _h}/{\text{ }}l)/2\,=\,{\Delta _h}/{\text{ }}l\,+\,\alpha /2$$

The load cell was moved manually during testing. So, additional LVDT was used to monitor the out-of-plane movement during testing to ensure that the load cell acting along the line of action passing through the center of out-of-plane direction of the beam. The out-of-plane movement was excluded in the calculations of chord rotations of the coupling beam.

**Fig. 9:**
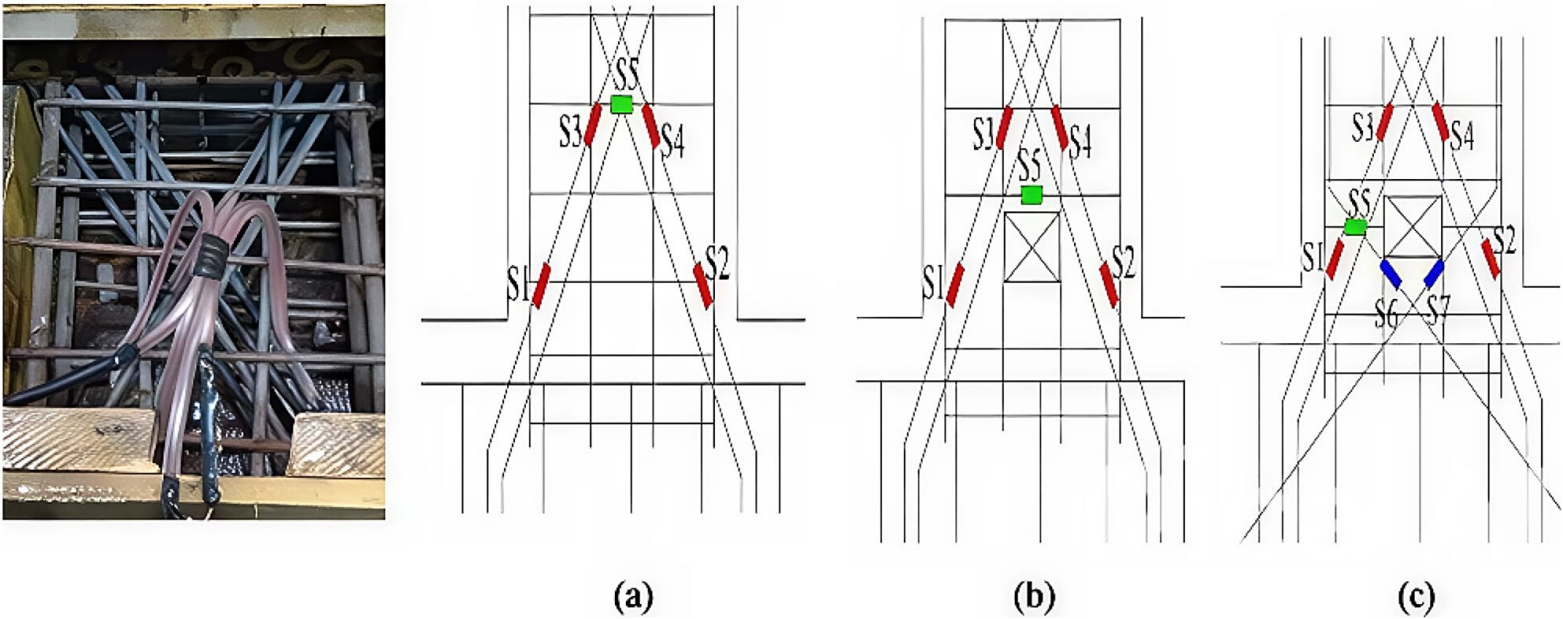
Strain gauges’ installation and locations: (a) Specimen (S); (b) Specimen (E-Op); and (c) specimen (E-Op-Ad).

To better understand the behavior of the specimens during testing, five strain gauges were installed in each specimen to measure strains at critical sections of the reinforcement bars. Four critical sections were chosen based on previous literature: two on the diagonal bars at the intersection of the two diagonal groups (at the beam mid-span) and two at the beam ends (adjacent to the fixed block). The fifth strain gauge was installed on the vertical stirrups adjacent to the opening, in the region expected to experience high shear stress concentration. For specimen E-Op-Ad, two additional strain gauges were added at the beam ends to measure the strain in the added diagonal bars and short stirrups. The locations of the strain gauges are shown in Fig. 9. The measured strain histories in the specimens were recorded and are presented in the Appendix A.

### Test results and discussions

The test results of the specimens, including crack propagation, failure modes, cracking loads, shear capacity, and cyclic curves (representing the relationship between chord rotation (θ) and shear force (V)), were extracted and discussed to compare the behavior of solid coupling beams with those containing openings.

### Initiation of cracks and cracking load

**Fig. 10:**
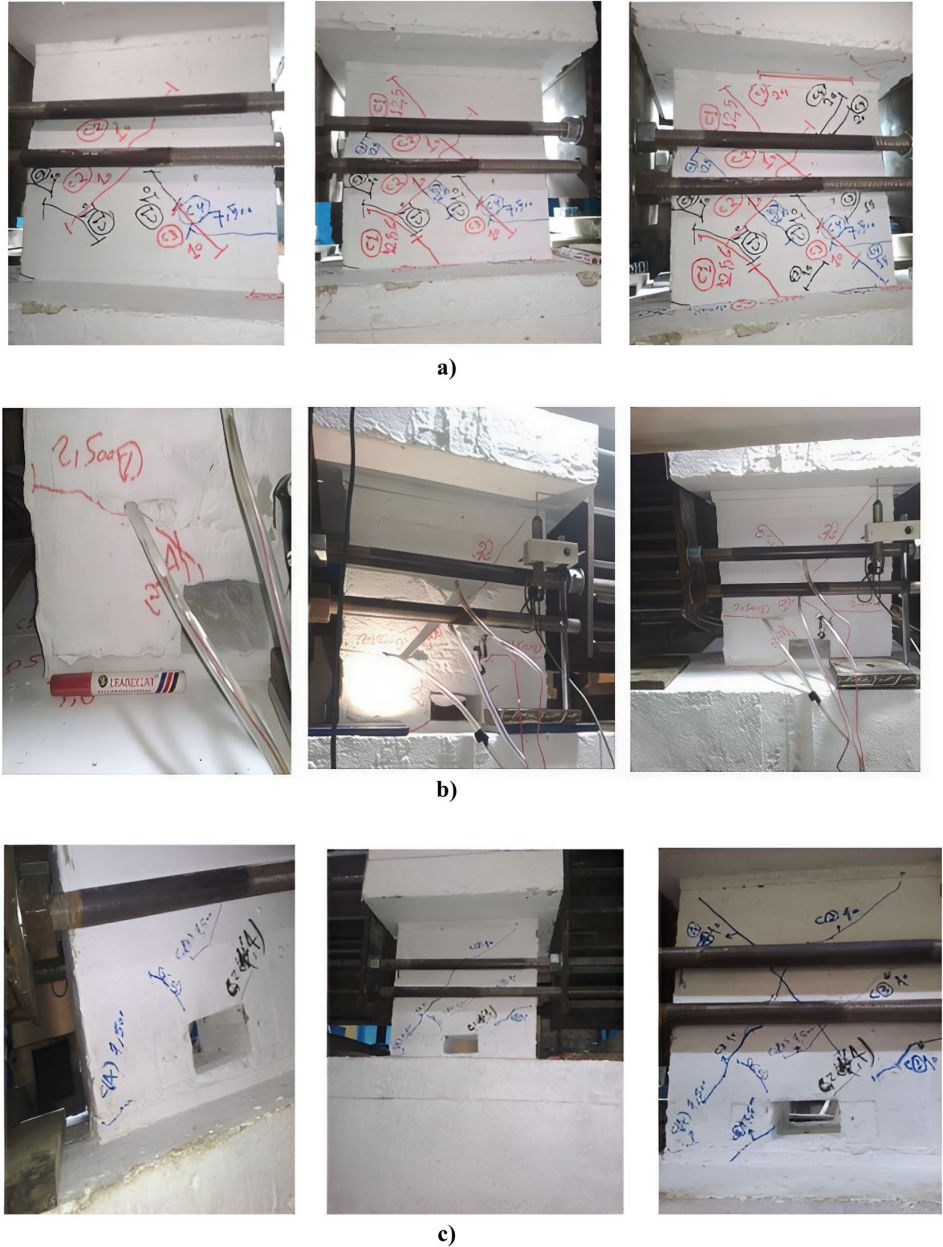
Pattern and propagation of cracks at different rate of loading for: (a) Specimen (S); (b) Specimen (E-Op); and (c) specimen (E-Op-Ad).

The initiation, location, shape of cracks, and type of failure were observed and discussed. For solid coupling beam, the first crack appeared near the mid-height of the beam at a load of P_cr_= 75 kN. This crack was followed by small diagonal cracks, these cracks grew very slowly and spread diagonally across the beam. At higher loading rates, these cracks grew significantly, increasing in both height and depth as the applied load increased. For the other two coupling beams with openings, the first crack initiated diagonally at the external top corner of the opening, immediately followed by small cracks at the beam end and small diagonal cracks originating from the opening’s ends and extending through the beam ends. Cracks at the corners of the opening increased and grew as the applied load increased. Figure 10 from a to c illustrates the pattern and propagation of cracks at different stages of loading during testing for the three specimens. The presence of openings decreased the cracking loads (P_cr_) to 25 kN and 40 kN for specimens E-Op and E-Op-Ad, respectively, representing reductions of 66.67% and 46.67% compared to the solid beam (S).

### Shear-rotation responses

**Fig. 11:**
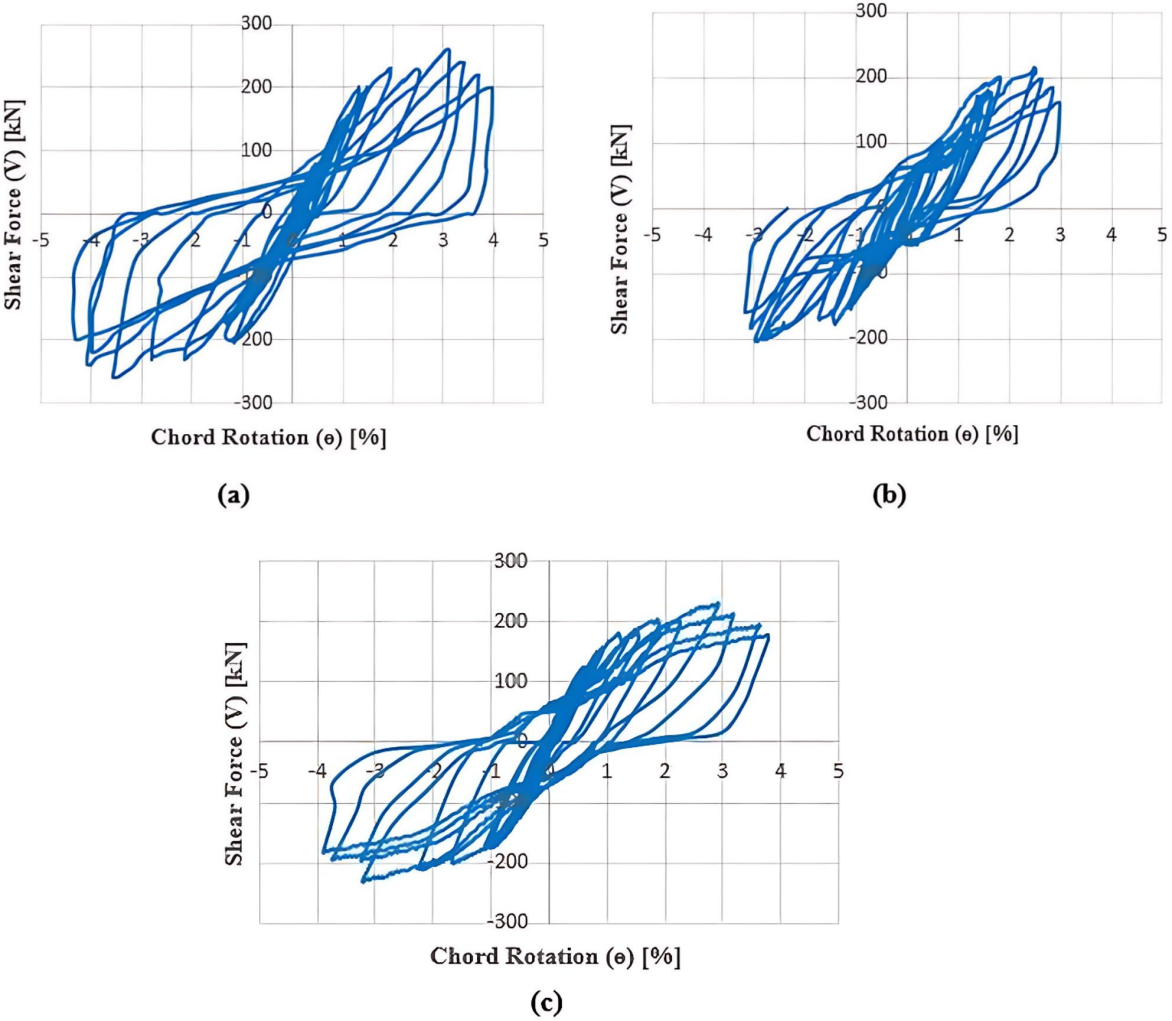
Cyclic curves of tested specimens: (a) (S); (b) (E-Op); and (c) (E-Op-Ad).

**Fig. 12:**
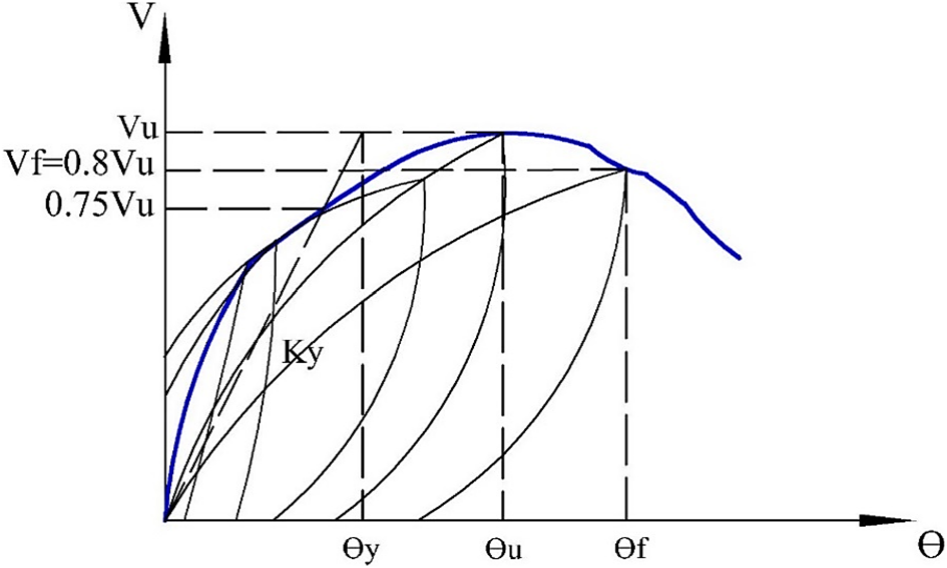
Main characteristics of the envelop curve.

The relationship between shear force (*V*) and rotation (*θ*) reveals the key characteristics of the seismic behavior of coupling beams, such as shear capacity, ductility, and stiffness degradation. Cyclic curves for all specimens are presented in Fig. 11 (a to c). The main parameters (*V*_*y*_, *V*_*u*_, *V*_*f*_, *θ*_*y*_, *θ*_*u*_, *θ*_*f*_, and *K*_*y*_), which represent the yield shear force, ultimate capacity, failure load, yield rotation, ultimate chord rotation, chord rotation at failure, and stiffness at the yield point, respectively, were extracted from the envelope curve, as illustrated in Fig. 12 and listed in Table 3. The yield point was determined according to^28^ as the intersection of a horizontal line representing the ultimate load and a straight line extending from the origin through a point representing 3/4 of the ultimate capacity. Vu is the peak strength (ultimate load), and *V*_*f*_ is defined as the point at which the strength has reduced by 20% of the ultimate value, *V*_*u*_. *θ*_*y*_, *θ*_*u*_, and *θ*_*f*_ represent the rotations at the yield point, ultimate load, and failure load, respectively.

**Table 3:** Main items of the hysteretic curve.

Specimen	V_y_ (kN)	V_u_ (kN)	θ_y_ (%)	θ_u_ (%)	θ_f_ (%)	µ	K_y_ (kN/rad)
S	221.86	259.1	1.96	3.12	3.96	2.02	11319.4
E-Op	195.3	217	1.85	2.51	2.98	1.61	10556.8
E-Op-Ad	204.6	239.7	1.93	2.92	3.85	1.99	10,601

### Shear capacity and progress of damage and failure

**Fig. 13:**
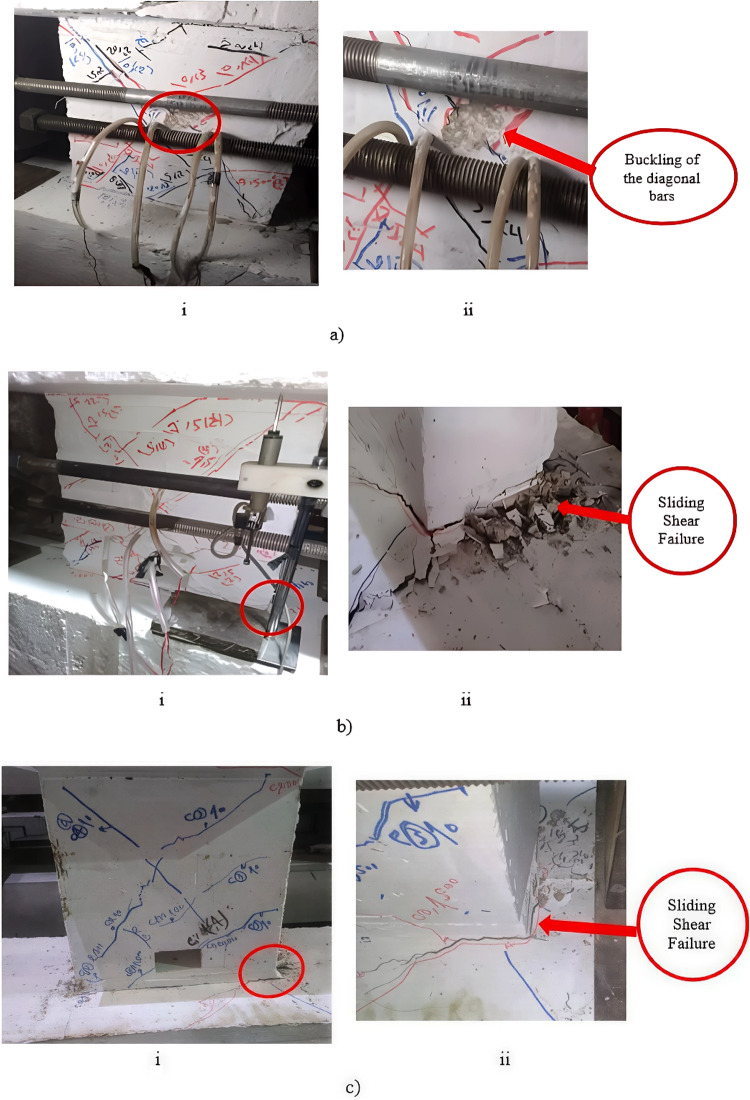
Damage of coupling beams, (i) Pattern of cracks at failure stage and (ii) mode of failure for specimens: (a) S, (b) E-Op, and (c) E-Op-Ad.

During testing, the evolution of damage was observed to investigate the behavior of different specimens at the final stage until failure. For the solid coupling beam (S), damage occurred as a result of the widening of two major diagonal cracks, which increased significantly as the loading rate increased. Simultaneously, a large, widening crack increased significantly at the end side of the beam. Failure ultimately occurred due to buckling of the diagonal bars and crushing of concrete in the diagonal direction of the coupling beam, accompanied by separation damage between the beam and the fixed block. In the case of the coupling beam with an end opening (E-Op), very extensive cracks spread along the entire length of the beam, especially around the opening, accompanied by a very wide crack at the beam end, which increased significantly as the loading rate increased, causing separation between the beam and the fixed block. Adding reinforcement around the opening in specimen (E-Op-Ad) effectively controlled the cracks around the opening. The cracking pattern was similar to that of the solid one, with two major diagonal cracks that increased significantly as the loading rate increased. However, at high loading rates (high rotations), the crack at the edge of the beam increased sharply, and the beam finally failed due to separation between the beam and the fixed block. This damage was accompanied by significant damage in the diagonal directions of the beam as a result of the widening of the two major diagonal cracks. Figure 13 (a to c) displays the deterioration and damage at the failure stage for the different specimens.

**Fig. 14:**
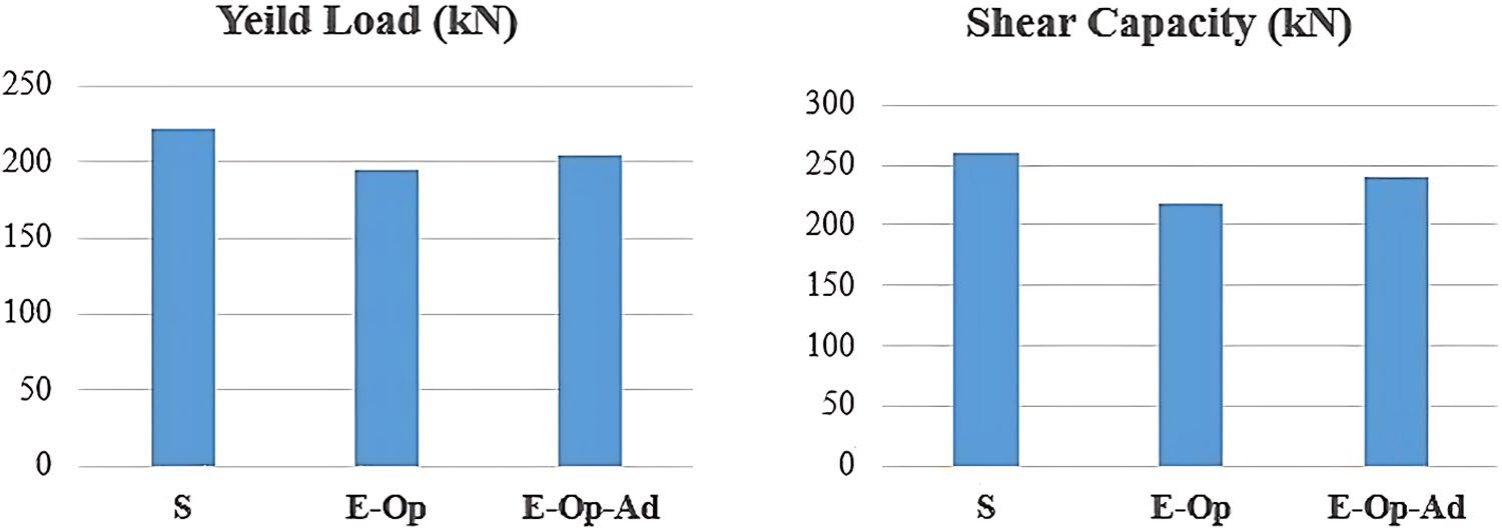
Yield and ultimate loads comparison between the tested specimens.

Based on the results in Table 3, a comparison was made in Fig. 14 between the solid coupling beam and the corresponding beams with openings. The results indicate that introducing an opening at the end of the coupling beam near the fixed block, specimen (E-Op), caused a reduction in the yield and ultimate shear forces of 11.97% and 16.25%, respectively. This effect decreased to 7.78% and 7.49% when intersecting diagonal bars and short stirrups were added around the opening in specimen (E-Op-Ad), which successfully mitigated the effect of the opening by decreasing the stress concentration around the opening. On the other hand, all the three specimens achieved maximum strength exceeded the shear capacity formula recommended by ACI^1^. In case of the standard beam (S) the maximum strength was higher than ACI’s formula by about 64%. However, for the other beams with openings (E-Op and E-Op-Ad) the increase reduced to 37.3% and 51.3%, respectively.

### Ductility

Ductility, the ability of a member to undergo large deformations before failure, is considered one of the most important characteristics for evaluating the suitability of coupling beams in resisting seismic loads in coupled shear wall systems. The ductility index (*µ*) is the most widely used method for quantifying ductility and can be calculated according to^28^ using the following equation:

7 $${{\mu }}={\theta _f}/{\theta _y}$$

where *θ*_*y*_ and *θ*_*f*_ are the yielding and maximum chord rotations, respectively, as described previously in Fig. 12. Table 3 lists the yield and maximum chord rotations for the different specimens. The solid coupling beam exhibited ductile behavior with µ = 2.02, achieving 1.96% and 3.96% for the yield rotation (*θ*_*y*_) and maximum chord rotation (*θ*_*f*_), respectively. The presence of the opening in specimen (E-Op) negatively impacted ductility, reducing *µ*, *θ*_*y*_, and *θ*_*f*_ to 1.61, 1.85%, and 2.98%, respectively. The reinforced coupling beam (E-Op-Ad) effectively controlled cracking and reduced stress concentration at the opening edges, resulting in improved performance compared to E-Op and exhibiting behavior similar to the solid beam (S), with slight decreases in *µ*, *θ*_*y*_, and *θ*_*f*_ to 1.99, 1.93%, and 3.85%, respectively.

### Stiffness degradation and yield stiffness

**Fig. 15:**
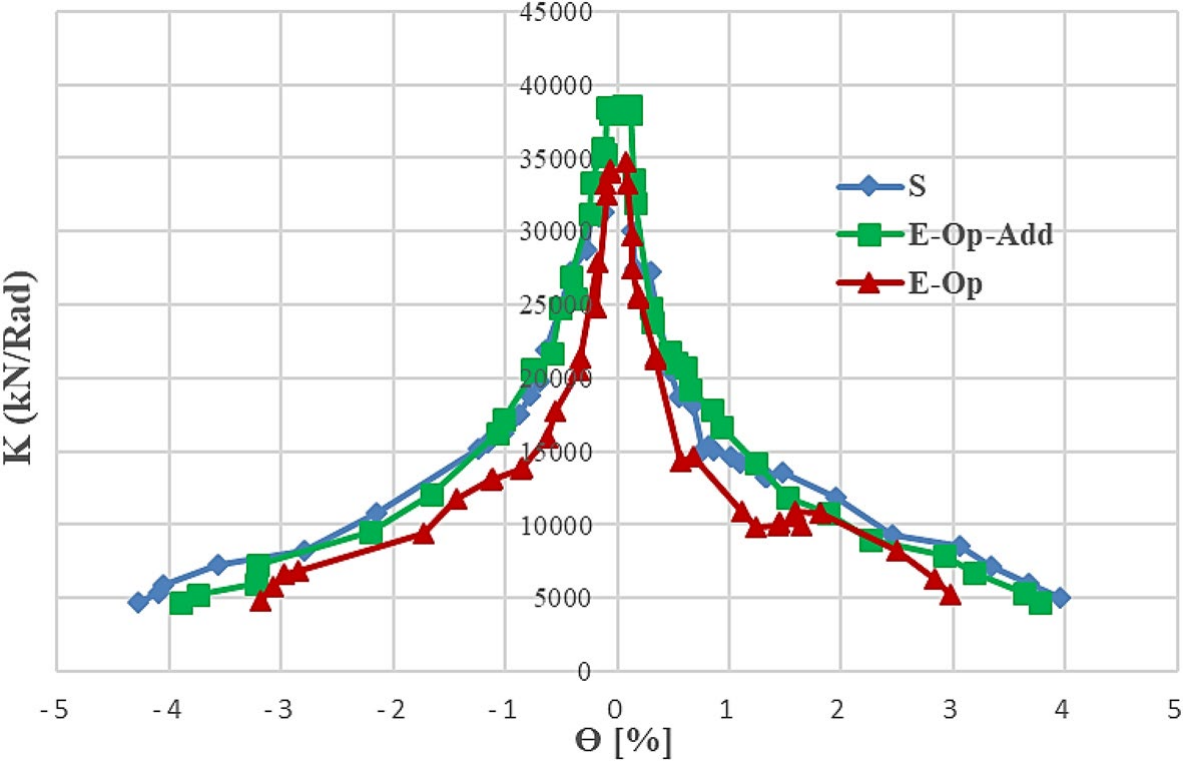
Stiffness degradation of tested specimens.

Analysis of stiffness reduction for reinforced concrete (RC) members subjected to cyclic loading is widely used to express damage deterioration and determine the suitability of members for resisting seismic loads. Members exhibiting sudden and high-rate stiffness reduction at low rotation percentages should be avoided in systems subjected to severe seismic loads and replaced with members exhibiting gradual and low stiffness reduction coupled with high ductility. Stiffness degradation can be extracted from the relationship between secant stiffness (*K*) and rotation (*θ*), where *K* is the secant stiffness at the amplitude of each half-cycle. Stiffness degradation for the different specimens was extracted and plotted in Fig. 15. The specimens (S) and (E-Op-Ad) exhibited gradual and low stiffness reduction compared to specimen (E-Op), which showed a significant reduction in stiffness as the rotation increased. Adding reinforcement detailing around the opening in specimen (E-Op-Ad) successfully improved the stiffness of the coupling beam and even surpassed that of the corresponding solid one at small rotations. However, it failed to control stiffness deterioration at large deformations. This is because the added reinforcement scheme around the opening controlled cracking, leading to improved stiffness in the early stages, but it could not prevent separation between the coupling beam and the block.

The yield stiffness is calculated by dividing the shear force by the corresponding rotation at the first yield point (see Fig. 12). For the solid specimen, the calculated yield stiffness was 11319.4 kN/rad. However, the yield stiffness for beam with opening specimens (E-Op and E-Op-Ad) was 10556.8 and 10,601 kN/rad, respectively. Thus, presence of small opening caused slight reduction in yield stiffness of about of only about 6% to 7% compared to the corresponding solid beam. This reduction was not appreciably affected by added special reinforcement.

### Energy dissipation

**Fig. 16:**
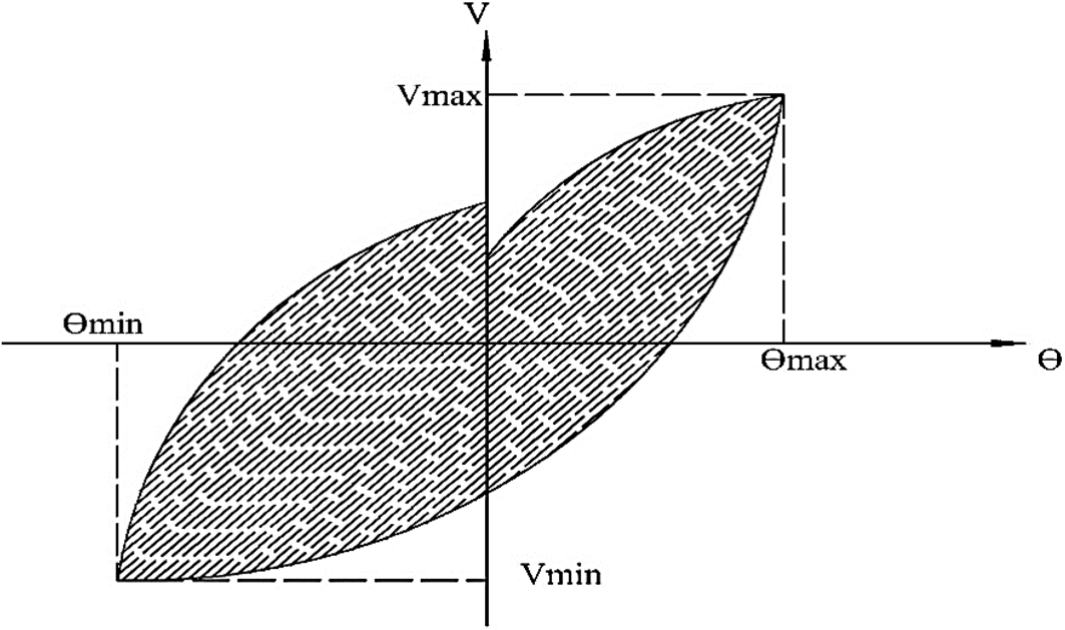
Area of energy dissipation at each load cycle.

**Fig. 17:**
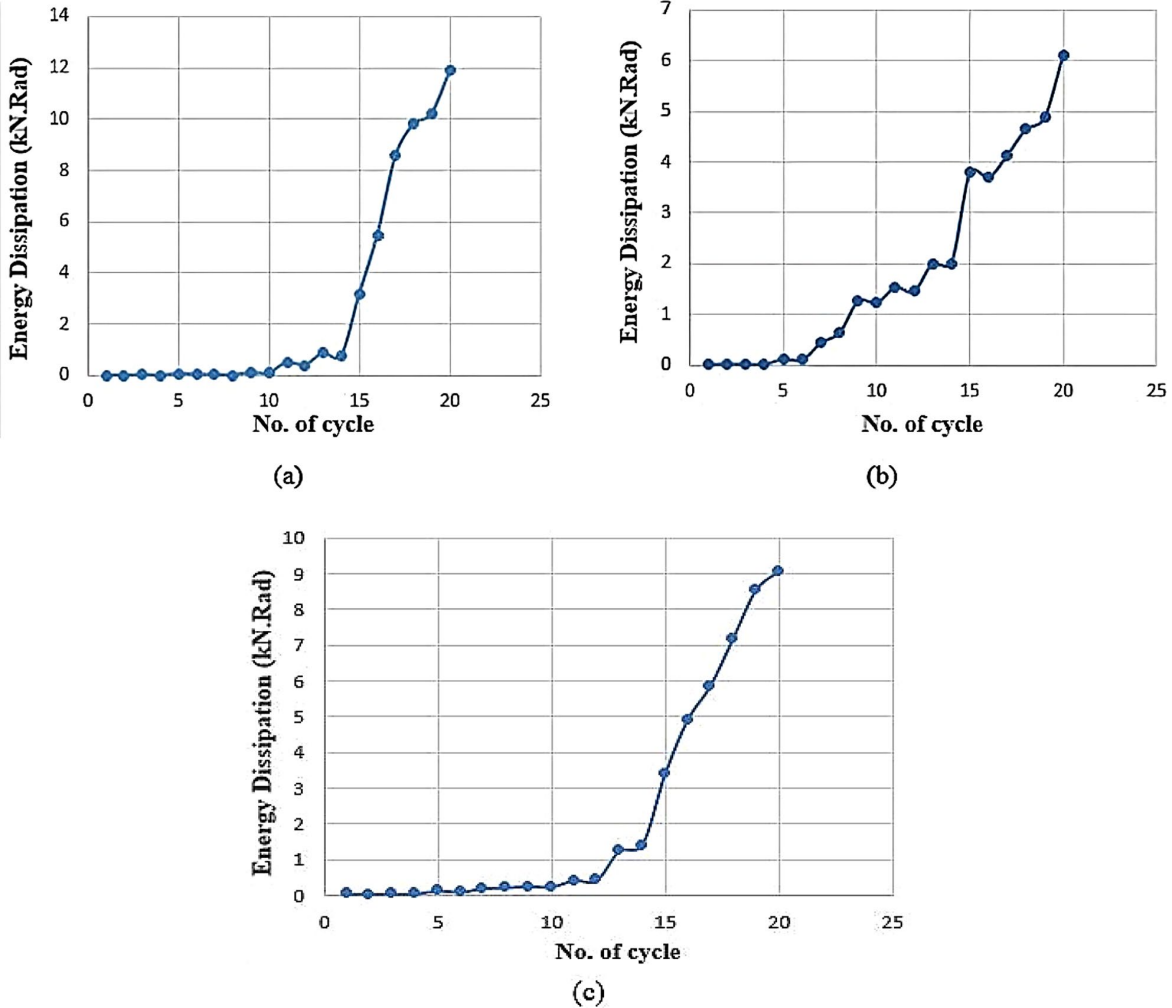
Energy dissipation at each load cycle: (a) Specimen (S); (b) Specimen (E-Op); and (c) specimen (E-Op-Ad).

**Fig. 18:**
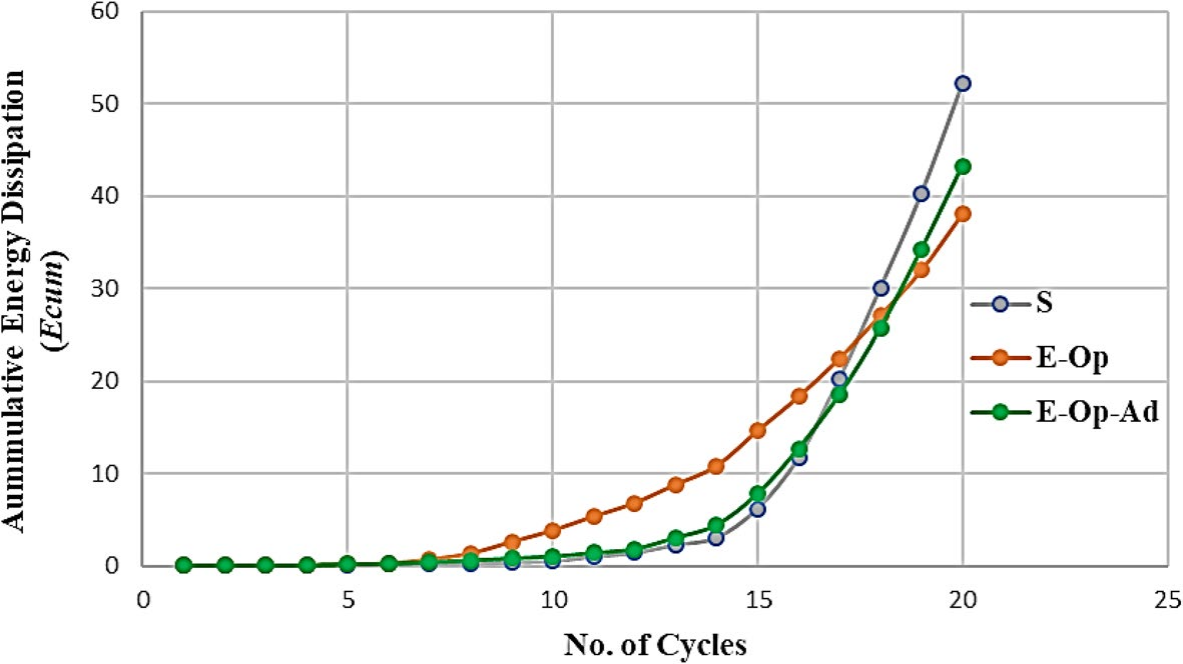
Accumulative energy dissipation (${E_{cum}}$) at each load cycle.

Energy dissipation plays an important role in members subjected to severe seismic loads. Energy dissipation is defined as the ability of a member to absorb energy and undergo large deformations without failure. It can be calculated by summing the area enclosed by the shear-rotation hysteresis loops at each cycle, as illustrated in Fig. 16. The energy dissipation was calculated at each load cycle for different specimens and plotted in Fig. 17 (a to c). The cumulative energy dissipation (*ΣE*), which is recommended for expressing energy dissipation behavior, was also calculated in this study and compared in Fig. 18. The figures indicate that CBs (S and E-Op-Ad) have approximately equal energy dissipation at each load cycle. Up to cycle No. 10, at (*V/Vu* = 0.48 and 0.52) for CBs S and E-Op-Ad, respectively, the two CBs dissipate small amounts of energy due to their higher stiffness, which results in smaller rotations. The energy dissipation increases slowly up to cycle No. 14, at (*V/Vu* = 0.68 and 0.73) for CBs S and E-Op-Ad, respectively. After this cycle, the dissipated energy increased sharply, indicating greater stiffness loss and thus larger rotations as the loading increased. The significant increase in energy dissipation appeared earlier in the case of CB (E-Op) at cycle No. 6, at (*V/Vu* = 0.35), and increased sharply up to the end of loading. The solid coupling beam (S) exhibited higher cumulative energy dissipation, equal to 52.18 kN·rad, surpassing the other CBs with openings (38.08 and 43.24 kN·rad), with reductions of approximately 27% and 17.1% for CBs (E-Op and E-Op-Ad), respectively, compared to the solid one.

### Comparative study with the results of^26^

This study and the work of^26^ both investigate the influence of rectangular openings on the behavior of diagonally reinforced coupling beams (CBs). However, key methodological differences likely account for the divergence in our findings.

The experimental program in^26^ utilized specimens with notably higher material strengths (${f_{cu}}$ = 38 MPa and ${f_y}$ = 510 MPa), greater reinforcement (4Ø16 diagonal bars with confinement stirrups at 11Ø10/m), a larger span-to-depth ratio (1.29), a wider beam width (200 mm), and smaller relative opening sizes (*l*_*o*_*/l*= 0.13 and *h*_*o*_*/t*= 0.17). Under these conditions^26^, concluded that two openings near the beam-wall interface had a negligible effect, with the perforated specimen demonstrating hysteretic behavior, shear capacity, and stiffness equivalent to its solid counterpart.

In contrast, the present study demonstrates that a single, larger opening (*l*_*o*_*/l* = 0.14 and *h*_*o*_*/t* = 0.24) in the same critical region significantly degrades the overall structural performance, as detailed in the previous sections. This discrepancy underscores the sensitivity of CB behavior to opening size and specific design parameters.

Therefore, based on our results, we recommend that if rectangular openings are necessary, their dimensions should not exceed the thresholds identified by^26^ (*l*_*o*_*/l* = 0.13 and *h*_*o*_*/t* = 0.17) to mitigate adverse effects. Further parametric studies are essential to comprehensively explore the influence of opening size, location, quantity, and reinforcement details to formulate robust design guidance.

## Conclusions

In this study, the effect of opening on the behavior of diagonally reinforced coupling beams, as per ACI^1^ second confinement option (no confinement around diagonal bars), is investigated. For this purpose, a limited-size experimental study consisting of three specimens (one solid and two with openings) is conducted. The behavior of CB with openings-specifically- pattern and propagations of cracks, yield and ultimate loads, ductility, stiffness degradation, and energy dissipation was investigated and compared with those of the solid beam. Based on results of the conducted experiments, it is found that:

- Presence of an opening with relative dimensions (*l*_*o*_*/l* = 0.14 and *h*_*o*_*/t* = 0.24) at beam near span end produced significant effects on the behavior of CB compared to the corresponding solid CB as it caused:
  1. Reduction in the yield strength, ultimate strength, and ductility by 11.97%, 16.25%, and 12.87%, respectively.
  2. Rapid deterioration of stiffness, it decreased the yield stiffness and the total dissipated energy by 6.73% and 27%, respectively.
  3. Increased the number of cracks especially at the opening corners, and changed the failure mode to sliding shear failure.

b) For the same opening size and location as in a), use of additional diagonal rebar plus short stirrups above and below the opening improved the overall beam behavior, namely it:
  1. Decreased the number of cracks and increased the strength, ductility, and total dissipated energy by 10.46%, 23.60% and 13.55%, respectively.
  2. Eliminated the effect of opening to a great deal as it yielded results close to the coupling beam without opening with reductions in strength, ductility, yield stiffness and total dissipated energy of only 7.5%, 1.49%, 6.3%, and 17%, respectively.

c) General:
  1. The experimental shear capacity of solid coupling beam (259.1 kN) is about 64% higher than that predicted by ACI-318 (158 kN). Thus, ACI-318 appears to be over-conservative.
  2. The use of half-scale models in coupling beam tests influences the results and their interpretation. Observed stress distribution and crack propagation patterns as well as derived ductility and energy dissipation values are attributed to this scale.
  3. Due to the limited scope of this study—which investigated only three specimens with a single opening configuration near the beam end—further research is recommended. Future work should explore a wider range of parameters to strengthen the applicability of these findings for design guidance.

## Supplementary Information

Below is the link to the electronic supplementary material.

Supplementary Material 1

## Data Availability

All data generated or analyzed during this study are included in this published article.

## Abbreviations

l: Length of the coupling beam
h: Height of the coupling beam
l_o_: Length of the opening
h_o_: Height of the opening
V_y_: Yield shear force
V_u_: Ultimate shear force
V_f_: Failure load
θ_y_: Rotation at yield shear force
θ_u_: Rotation at ultimate shear force
θ_f_: Rotation at failure load
K_y_: Stiffness at the yield point
µ: Ductility
CBs: Coupling beams
RFT: Reinforcement

## References

[1] ACI Committee 318. *Building Code Requirements for Structural Concrete (ACI 318-19) and Commentary (ACI 318R-19)* (American Concrete Institute, 2019). 10.14359/51716937.

[2] Kwan, A. K. H. & Zhao, Z-Z. Cyclic behaviour of deep reinforced concrete coupling beams. *Proc. Inst. Civ. Eng.-Struct. Build.* **15**, 283–293. 10.1680/stbu.2002.152.3.283 (2002).

[3] Galano, L. & Vignoli, A. Seismic behavior of short coupling beams with different reinforcement layouts. *Struct. J.* **97**, 876–885. 10.14359/9633 (2000).

[4] Paulay, T. & Binney, J. R. *Diagonally Reinforced Coupling Beams of Shear Walls, American Concrete Institute* 579–598 (ACI Symposium Publication, 1974).

[5] Lim, E., Hwang, S. J., Wang, T. W. & Chang, Y. H. An investigation on the seismic behavior of deep reinforced concrete coupling beams. *ACI Struct. J.* **113** (2), 217–226. 10.14359/51687939 (2016).

[6] Lim, E., Hwang, S. J., Cheng, C. H. & Lin, P. Y. Cyclic tests of reinforced concrete coupling beam with intermediate Span-Depth ratio. *ACI Struct. J.* **113** (3), 515–524. 10.14359/51688473 (2016).

[7] Chou, T. A. Experimental and analytical studies of reinforced concrete coupling beams under seismic and wind loads. *Diss.*10.10371/209894 (2024).

[8] Paulay, T. & Nigel Priestley, M. J. *Seismic Design of Reinforced Concrete and Masonry Buildings* (Wiley, 1992). 10.1002/9780470172841.

[9] Barney, G. B. et al. *Behavior of Coupling Beams under Load Reversals (RD068.01B)* (Portland Cement Association, 1980). https://www.concrete.org/publications/internationalconcreteabstractsportal.aspx?m=details&ID=16830.

[10] Cheng, M. Y., Gitomarsono, J. & Hung-Yu, Z. Cyclic test of diagonally reinforced concrete coupling beam with different shear demand. *ACI Struct. J.* **116** (6), 241–250. 10.14359/51718010 (2019).

[11] Yang, C. et al. Behaviour and detailing of coupling beams with high-strength materials. *J. Build. Eng.* **47**, 103843. 10.1016/j.jobe.2021.103843 (2022).

[12] Han, S., Whan, S. B., Kim & Kim, T. Effect of transverse reinforcement on the seismic behavior of diagonally reinforced concrete coupling beams. *Eng. Struct.* **196**, 109307. 10.1016/j.engstruct.2019.109307 (2019).

[13] Park, W. S., Kang, T. H. K., Kim, S. & Yun, H. D. Seismic performance of moderately short concrete coupling beams with various reinforcements. *ACI Struct. J.* **117** (3), 141–154. 10.14359/51723501 (2020).

[14] Naish, D., Fry, A., Klemencic, R. & Wallace, J. W. Reinforced concrete coupling beams-—part I: testing. *ACI Struct. J.* **110** (6), 1057–1066. 10.14359/51686160 (2013).

[15] ASCE. *Seismic Evaluation and Retrofit of Existing Buildings (ASCE 41 -17) *(American Society of Civil Engineers (ASCE), 2017). 10.1061/9780784414859.

[16] Hindi, R. A. & Midhat, A. H. Shear capacity of diagonally reinforced coupling beams. *Eng. Struct.* **26**, 1437–1446. 10.1016/j.engstruct.2004.05.012 (2004).

[17] Han, S., Whan, J. W., Kang, C. & Hee, H. Shear strength equation for slender diagonally reinforced coupling beam. *J. Earthq. Eng. Soc. Korea* **20**, 361–368. 10.5000/EESK.2016.20.6.361 (2016).

[18] Gwon, S., Shin, M. & Lee, D. Nonlinear modeling parameters of RC coupling beams in a coupled wall system. *Earthq. Struct.* **7** (5), 817–842. 10.12989/eas.2014.7.5.817 (2014).

[19] Ding, R., Tao, M. X., Nie, X. & Mo, Y. L. Fiber beam-column model for diagonally reinforced concrete coupling beams incorporating shear and reinforcement slip effects. *Eng. Struct.* **153**, 191–204. 10.1016/j.engstruct.2017.10.035 (2017).

[20] Eom, T. S., Lee, S. J., Kang, S. M. & Park, H. G. Nonlinear modeling parameters of reinforced concrete coupling beams. *ACI Struct. J. Am. Concrete Inst.* **119** (2), 89101. 10.14359/51734139 (2022).

[21] Hwang, H. J., Kim, S. H., Kim, S. H., Park, M. I. & Park, H. G. Shear strength degradation model for performance-based design of short coupling beams. *ACI Struct. J.* **119** (4), 277–289. 10.14359/51734524 (2022).

[22] Hajyalikhani, P. Experimental study on seismic performance of reinforced concrete coupling beams and rectangular squat walls with innovative reinforcement configurations. *Diss.*. https://mavmatrix.uta.edu/cgi/viewcontent.cgi?article=1400&context=civilengineering_theses (2015).

[23] Seo, S. Y., Yun, H. D. & Young-Soo, C. Hysteretic behavior of conventionally reinforced concrete coupling beams in reinforced concrete coupled shear wall. *Int. J. Concrete Struct. Mater.* **11**, 599–616. 10.1007/s40069-017-0221-8 (2017).

[24] Dajun, D., Zhengliang, C. & Zhang, S. Experimental studies of new ductile coupling beams and multi-storey shear walls. *Mater. Struct.* **30**, 566–573. 10.1007/BF02486402 (1997).

[25] Choi, Y., Hajyalikhani, P. & Chao, S. H. Seismic performance of innovative RC coupling beam—Double-Beam coupling beam. *ACI Struct. J.* **115** (1), 113–125. 10.14359/51700951 (2018).

[26] Sayed, A., El-Azim, H. A., Haggag, J. & Wael, M. H. Performance of coupling beams with openings exposed to Cyclic loading. *Eng. Res. J.* **168**, 191–210. 10.21608/erj.2020.140846 (2020).

[27] Mansur, T. M. A. Design of reinforced concrete beams with web openings. In *Proceedings of the 6th Asia-Pacific structural Engineering and Construction Conference (ASPEC 2006). Malaysia: Kuala Lumpur*. https://core.ac.uk/download/pdf/11777413.pdf (2006).

[28] Pan, A. & Moehle, J. P. Lateral displacement ductility of reinforced concrete flat plates. *Struct. J.* **86** (3), 250–258. 10.14359/2889 (1989).

