# Supplementary material for: Experimental evaluation of the effect of openings on the structural performance of diagonally reinforced coupling beams
Source: Sci Rep. 2025 Dec 15;15:43867. doi: 10.1038/s41598-025-25787-7 (PMC12708849; doi:10.1038/s41598-025-25787-7)
Supplement: Supplementary file 1 — Supplementary Material 1 [file 41598_2025_25787_MOESM1_ESM.docx]

**Appendix A:** Measured strain histories in tested specimens

1. b)

c)

Figure A.1 Measured strain histories in specimen S: (a) Strain 1 (S); (b) Strain 2; and (c) Strain3.

1. b)

c) d)

Figure A.2 Measured strain histories in specimen E-Op: (a) Strain 1 (S); (b) Strain 3; (c) Strain 4; and (d) Strain5.

1. **b)**

**c) d)**

**e)**

Figure A.3 Measured strain histories in specimen E-Op-Ad: (a) Strain 1 (S); (b) Strain 2; (c) Strain 5; (d) Strain 6; and (e) Strain7.
